# Assessment and management of patients detained under Section 136 in Northwick Park Hospital emergency department

**DOI:** 10.1192/bjo.2021.935

**Published:** 2021-06-18

**Authors:** Laurence Telesia, Lauren Fraser

**Affiliations:** 1 South London and Maudsley NHS Foundation Trust; 2 Northwick Park Hospital, London North West University Healthcare NHS Trust

## Abstract

**Aims:**

To evaluate the role of the Emergency Medicine team (EM) within a London Emergency Department (ED) in assessing and managing patients detained under Section 136 of the Mental Health Act, 1983 (S136).

**Background:**

S136 allows detention and transfer of people to ED and psychiatric hospitals for further assessment. EDs are optimised for the investigation and management of the medically unwell, but attending ED may also delay access to psychiatric services if required. Minimal research has been performed to investigate the relative benefits of transferring people under S136 to ED versus psychiatric hospitals.

**Method:**

Electronic notes were searched to identify those attending under S136 between 01/04/2017 and 31/03/2018. Scanned medical notes were reviewed and data extracted regarding patient demographics, length of ED stay, reason for S136 use, investigations and interventions undertaken by EM.

**Result:**

This identified 95 attendances by 87 patients. The mean age was 35 years (range 15-75) and 59% of attenders were male. The mean duration of stay was 7 hours 34 minutes (range 6 minutes - 25 hours 50 minutes).

Reasons for S136 use were abnormal behaviour (32), expressed suicidal ideation (29), overdose (15), self-harm (13), overdose plus self-harm (4), being found wandering (1) and was unclear for 1 presentation.

In 39 attendances no investigations beyond history and examination were performed by EM. Only 6 patients had investigations that were not bloods, electrocardiogram or urinalysis. These included X-radiograph trunk (4), computed tomography (CT) head (3), X-radiograph limb (3), CT cervical spine (1), Focused Assessment with Sonography for Trauma (1).

No interventions were given by EM in 55 attendances. Twenty-nine different medications were prescribed and 18 patients were prescribed intravenous fluids. Three had wounds dressed, 3 glued, 3 sutured and 1 stapled.

**Conclusion:**

There were difficulties categorising the reason for S136 use, as clear documentation was often unavailable, but the vast majority of patients were detained due to abnormal behaviour, expressed suicidal ideation and self-harm.

Few attending ED under S136 received investigations or interventions that could not be offered within a psychiatric hospital. There was a wide range in duration of stay within ED, however 65% of attendances were longer than the standard 4 hour target.

Future research may assess the relative benefits of ED versus psychiatric hospitals in assessing those detained. This could aid services in meeting both the physical and psychiatric needs of patients whilst making efficient use of available resources.

